# Mechanism-Specific Pharmacodynamics of a Novel Complex-I Inhibitor Quantified by Imaging Reversal of Consumptive Hypoxia with [^18^F]FAZA PET *In Vivo*

**DOI:** 10.3390/cells8121487

**Published:** 2019-11-21

**Authors:** Seth T. Gammon, Federica Pisaneschi, Madhavi L. Bandi, Melinda G. Smith, Yuting Sun, Yi Rao, Florian Muller, Franklin Wong, John De Groot, Jeffrey Ackroyd, Osama Mawlawi, Michael A. Davies, Y.N. Vashisht Gopal, M. Emilia Di Francesco, Joseph R. Marszalek, Mark Dewhirst, David Piwnica-Worms

**Affiliations:** 1 Department of Cancer Systems Imaging, The University of Texas MD Anderson Cancer Center, Houston, TX 77030, USA; stgammon@mdanderson.org (S.T.G.); FPisaneschi@mdanderson.org (F.P.); YRao@mdanderson.org (Y.R.); FMuller@mdanderson.org (F.M.); Jeffrey.Ackroyd@uth.tmc.edu (J.A.); 2 Translational Research to Advance Therapeutics and Innovation in Oncology, The University of Texas MD Anderson Cancer Center, Houston, TX 77030, USA; mbandi06@gmail.com (M.L.B.); MGSmith@mdanderson.org (M.G.S.); YSun8@mdanderson.org (Y.S.); JRMarszalek@mdanderson.org (J.R.M.); 3 Department of Nuclear Medicine, The University of Texas MD Anderson Cancer Center, Houston, TX 77030, USA; fwong@mdanderson.org; 4 Department of Neuro-Oncology, The University of Texas MD Anderson Cancer Center, Houston, TX,77030, USA; jdegroot@mdanderson.org; 5 Department of Imaging Physics, The University of Texas MD Anderson Cancer Center, Houston, TX 77030, USA; omawlawi@mdanderson.org; 6 Department of Melanoma Medical Oncology, The University of Texas MD Anderson Cancer Center, Houston, TX 77030, USA; MDavies@mdanderson.org (M.A.D.);; 7 Institute for Applied Cancer Science, The University of Texas MD Anderson Cancer Center, Houston, TX 77030, USA; MEDiFrancesco@mdanderson.org; 8 Department of Radiation Oncology, Duke University School of Medicine, Durham, NC 27710, USA; mark.dewhirst@duke.edu

**Keywords:** hypoxia, [^18^F]FAZA, PET, IACS-010759, mitochondrial complex I, metabolism, pharmacodynamics, oxidative phosphorylation, collateral lethality

## Abstract

Tumors lack a well-regulated vascular supply of O_2_ and often fail to balance O_2_ supply and demand. Net O_2_ tension within many tumors may not only depend on O_2_ delivery but also depend strongly on O_2_ demand. Thus, tumor O_2_ consumption rates may influence tumor hypoxia up to true anoxia. Recent reports have shown that many human tumors in vivo depend primarily on oxidative phosphorylation (OxPhos), not glycolysis, for energy generation, providing a driver for consumptive hypoxia and an exploitable vulnerability. In this regard, IACS-010759 is a novel high affinity inhibitor of OxPhos targeting mitochondrial complex-I that has recently completed a Phase-I clinical trial in leukemia. However, in solid tumors, the effective translation of OxPhos inhibitors requires methods to monitor pharmacodynamics in vivo. Herein, ^18^F-fluoroazomycin arabinoside ([^18^F]FAZA), a 2-nitroimidazole-based hypoxia PET imaging agent, was combined with a rigorous test-retest imaging method for non-invasive quantification of the reversal of consumptive hypoxia in vivo as a mechanism-specific pharmacodynamic (PD) biomarker of target engagement for IACS-010759. Neither cell death nor loss of perfusion could account for the IACS-010759-induced decrease in [^18^F]FAZA retention. Notably, in an OxPhos-reliant melanoma tumor, a titration curve using [^18^F]FAZA PET retention in vivo yielded an IC_50_ for IACS-010759 (1.4 mg/kg) equivalent to analysis ex vivo. Pilot [^18^F]FAZA PET scans of a patient with grade IV glioblastoma yielded highly reproducible, high-contrast images of hypoxia in vivo as validated by CA-IX and GLUT-1 IHC ex vivo. Thus, [^18^F]FAZA PET imaging provided direct evidence for the presence of consumptive hypoxia in vivo, the capacity for targeted reversal of consumptive hypoxia through the inhibition of OxPhos, and a highly-coupled mechanism-specific PD biomarker ready for translation.

## 1. Introduction

Fundamentally, the concentration of intracellular oxygen in tumor cells is the net result of the rate at which oxygen (O_2_) diffuses into cells and the rate at which oxygen is consumed [1], and tumor hypoxia results from a mismatch in the diffusion of O_2_ relative to the consumption rate [1,2]. Thus, diffusional hypoxia in tumors is generally attributed to O_2_ supply, such as low blood O_2_ tension (hypoxemic hypoxia); reduced capacity of blood to carry O_2_ (anemia, methemoglobin, or carbon monoxide; anemic hypoxia); and reduced tissue perfusion through disorganized tumor blood vessels and deterioration of diffusion geometries, e.g., heterogeneous diffusion distances, and concurrent versus countercurrent blood flow within microvessels [3,4,5]. Because of finely tuned regulatory processes in normal tissues, increases in the tissue O_2_ consumption rate (OCR) are generally matched by an increase in blood flow and, therefore, do not usually lead to hypoxia. However, in the context of neuronal activation and injury, the systems regulating blood flow can fail to meet the increased O_2_ demand of the tissue in question, leading Scholz et al. to coin the phrase “consumptive hypoxia” [6]. Indeed, the tumor microenvironment may be naturally susceptible to consumptive hypoxia, wherein the O_2_ consumption rate has been shown to be a highly significant parameter that strongly influences the extent and magnitude of tumor hypoxia [7,8,9,10,11,12,13]. The biochemical details of how metabolic reprogramming and O_2_ consumption rates impact tumor hypoxia in vivo remain under intense study [14,15].

There exists an extensive study in support of a shift in intermediate metabolism away from OxPhos in tumors [16], the conventional Warburg model, but much of these data are derived from indirect evidence, measurements of tumor cells in culture, and xenograft model systems. Recent analyses of metabolic flux in human tumors in vivo have challenged various details of conventional assessments of cancer metabolism [17,18,19]. For example, in living patients with non-small cell lung cancer (NSCLC) infused with ^13^C-labeled substrates in vivo, there is no evidence for suppressed glucose oxidation, and data instead suggest *activated* glucose oxidation relative to adjacent normal lung [17,18,19]. Furthermore, for a majority of tumors in these studies and contrary to expectations for aerobic glycolysis (Warburg), lactate is overall *imported*, and often used as the predominant fuel for the tricarboxylic acid (TCA) cycle [15,17,20]. These human studies further demonstrate robust glucose oxidation through TCA cycle flux, which was observed across diverse histological subtypes and oncogenotypes of NSCLC [17,18]. Overall, evidence from human NSCLC in vivo would support that oxidative phosphorylation (OxPhos) is a dominant energy generator with redox equivalents being derived from multiple carbon sources (glucose, lactate, glutamine, other amino acids) [17,18]. Thus, while tumor hypoxia was not directly determined in these human studies, an emerging isotopic flux analysis from tumors in living patients demonstrates that OxPhos is surprisingly active in many tumors, implying a significant oxygen consumption rate to meet metabolic demands and the potential for consumptive hypoxia to be more prevalent in tumors than previously presumed.

These clinical studies also complement recent reports that emphasize the complexity of tumor metabolic reprogramming; not only do tumor cells *utilize* OxPhos, but some also *depend upon* OxPhos for both energy and anabolism [21,22,23,24,25,26,27,28,29]. In this regard, IACS-010759 was developed to target OxPhos-dependent tumor cells. This novel compound targets mitochondrial complex-I to inhibit oxidative phosphorylation at nanomolar concentrations with highly effective pharmacokinetic properties [30]. As expected by the above model, in preclinical models of solid tumors, IACS-010759 mediated reversal of hypoxia in vivo was validated as a PD biomarker, but this validation was conducted by invasive pimonidazole-based staining. While an pimonidazole-IHC analysis may suffice for preclinical pharmacodynamic studies, for human solid tumor trials, the capacity to document and spatially map the IACS-010759-induced decrease in OCR and resulting reversal of consumptive hypoxia in patients within deep tissue sites is lacking.

**Figure 1:**
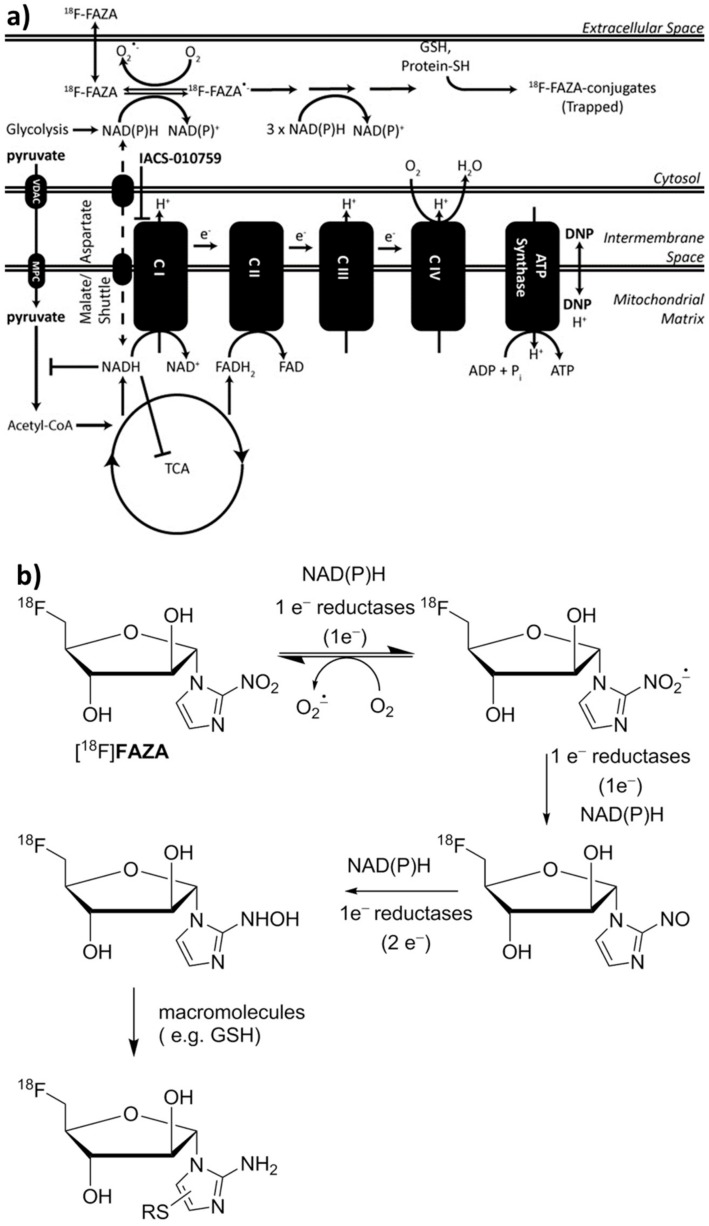
[^18^F]FAZA yields a mechanism-based PD readout of complex-I inhibitor IACS-010759. (**a**) Proposed mechanism of [^18^F]FAZA retention in relation to ETC inhibition. (**b**) The first step in the reduction of the nitro group can be reversed by O_2_ or free radicals. However, in hypoxic/highly reducing environments, the 2-nitroimidazole moiety can be further reduced, eventually reacting covalently with thiols, therefore trapping the radiolabeled probe in the cell. Indeed, determination of the intracellular redox potential of live cells can be derived biochemically from the free to oxidized thiol ratio, particularly from glutathione as the GSH:GSSG ratio, further linking the mechanism of trapping of [^18^F]FAZA to intracellular redox potential. In hypoxic conditions, both excess NAD(P)H and reduced forms of glutathione (GSH) increase the retention of these reporters [33].

Hypoxic conditions are ideal for trapping 2-nitroimidazole-based imaging reporters, such as ^18^F-labeled fluoroazomycin arabinoside ([^18^F]FAZA) (Figure 1a), which are sequentially reduced by NAD(P)H-dependent intracellular reductases in a manner tightly coupled to intracellular O_2_ content (Figure 1b) and ultimately conjugated to free thiols within cells, e.g., glutathione (GSH), to generate positron emission tomography (PET) images [31,32].

Herein, we provide direct evidence in vivo that inhibition of OCR by IACS-010759, a potent and specific drug candidate, robustly and rapidly relieved tumor hypoxia as predicted by quantitative mathematical models of consumptive hypoxia [13]. Because of the tight coupling between mitochondrial OCR and consumptive hypoxia, we demonstrated that in living animals [^18^F]FAZA PET can serve as a quantitative PD biomarker in vivo of IACS-010759. Furthermore, a proof-of-principle clinical study of the precision of [^18^F]FAZA PET for imaging hypoxia in a test-retest study of a patient with glioblastoma informed the pathway forward to a full human analysis.

## 2. Materials and Methods

### 2.1. In Vitro Analysis of Oxygen Consumption Rate

Cells were seeded on XF96 Cell Culture Microplates (Seahorse Bioscience, North Billerica, Billerica, MA, USA) in their original growth media 24 h prior the experiment, tumor cells at 20,000 cells per well and HPNE at 15,000 cells per well. To measure oxygen consumption rate (OCR), cells were washed and maintained with XF base medium lacking NaHCO_3_ and supplemented with 1 mM pyruvate (Sigma-Aldrich, St. Louis, MO, USA), 2 mM glutamine and 10 mM glucose, 10% dialyzed FBS and adjusted to pH 7.4. Using a Seahorse XF96 analyzer, four measurements were taken as basal OCR. Serial dilutions of IACS-010759 in XF base medium were injected to a final concentration of 0–300 nM and four repeat OCR measurements were taken 1 h after the drug injection.

### 2.2. [^18^F]FAZA Production and IACS-010759 Formulation

Using an automated synthetic module (GE Tracerlab), [^18^F]FAZA was produced from 1-(2,3-diacetyl-5-tosyl-(α-d-arabinofuranosyl)-2-nitroimidazole (ABX, Germany), following a procedure reported in the literature [34,35]. Purification was performed using a Luna C18(2) 5 μm 250 mm × 10 mm semi-preparative HPLC column. Radiochemical and UV purities were assessed by analytical HPLC using an Econosil C18 10 μm 250 mm × 4.6 mm column. The HPLC method for quality control was: (A) 10 mM NaH_2_PO_4_, (B) MeCN; %B: 5 for 3 min, 5 to 90 in 12 min, 90 to 5 in 2 min, 5 for 3 min. [^18^F]FAZA retention time was 10.0 min. [^18^F]FAZA was obtained in 50 min, with >99% radiochemical purity, up to 80 GBq/µmol molar activity and in 28.1 ± 3.8% (*n* = 37) activity yield (non-decay corrected) from aqueous fluoride. For human imaging, [^18^F]FAZA was similarly produced under GMP conditions in a clinical radiopharmacy.

IACS-010759 was synthesized as indicated previously and formulated for oral delivery in 0.5% methylcellulose [30]. The formulated drug and vehicle were stored at 4 °C and stirred continuously prior to use.

### 2.3. Animal Models

Female nude (*nu/nu*; Charles River) mice were maintained in an AAALAC-approved 12-h light-dark cycle facility and could eat/drink *ad libitum*. SK-MEL-5, A375, A375-R1, H460 tumor cell lines were implanted subcutaneously and grown to >100 mm^3^ in nude mice prior to PET/CT imaging. SK-MEL-5 and A375 cells were acquired from the MDACC characterized cell line core. A375-R1 are a drug-resistant derivative of A375 cells (ATCC) [29]. H460 cells were acquired from the ATCC. D423-Fluc cells were constructed from D423 cells using Cignal Lenti Positive Control kits according to the manufacturer’s instructions, passing mycoplasma and cell identity tests by the characterized cell line core. D423-Fluc cells were implanted orthotopically in female NOD/SCID gamma mice (NSG; Jackson Laboratory) by utilizing an intracranial bolt and monitored by MRI. Prior to PET/CT imaging tumors grew between 7 and 9 weeks post implantation and were > 20 mm^3^. Mouse care, treatments, and imaging were conducted under The University of Texas MD Anderson Cancer Center Institutional Animal Care and Use Committee (IACUC) protocols 00000884 and 00001179 and followed all appropriate regulations and laws.

### 2.4. [^18^F]FAZA Imaging and Analysis

Generally, mice were each imaged twice, baseline (Day 0) and 24 h post-treatment (Day 1). Mice were briefly anaesthetized (<5 min) using 1% to 2% isoflurane with O_2_ as a carrier. Mice were bolus injected i.v. with [^18^F]FAZA in sterile saline with a target of 11 MBq (300 μCi) per mouse in 150 µL of volume. Actual injected dose was calculated based on measuring the pre- and post-injection activity in the syringe with a dose calibrator (Capintec). Mice were then returned to their cages, became ambulatory quickly, and were allowed to eat and drink *ad libitum* for ~2 h 45 min. Mice were then re-anaesthetized using 1% and 3% isoflurane, transferred to a pre-clinical PET/SPECT/CT system (Albira PET/SPECT/CT, Bruker) and maintained at 0.5% to 2% isoflurane with continuous monitoring of respiration during the acquisition. PET images were acquired for 10 min using a 15 cm FOV centered on the tumor; CT images were acquired for fusion using a 7 cm FOV also centered on the tumor. After the initial imaging session, mice were treated with vehicle control (saline i.v., PBS i.t., or methylcellulose in saline p.o.) or IACS-010759 at the indicated dose p.o., and 24 h later injected a second time with tracer, and reimaged. The mice treated with IACS-010759 were particularly sensitive to isoflurane and were monitored carefully during image acquisition.

To determine if IACS-010759 significantly changed the initial uptake of [^18^F]FAZA, imaging was modified to include a 10 min PET/CT dynamic scan immediately after injection of tracer and then, mice were allowed to awaken and freely move about their cages until the 3 h time point for standard [^18^F]FAZA scanning.

Images were reconstructed using MLEM and FBP for PET and CT, respectively, and automatically fused by the software. Data were corrected for decay, randoms, and dead time using standard manufacturer-supplied software (Albira, Bruker), and expressed as %ID/cc (PMOD, PMOD Technologies). Tumor VOIs were constructed using the CT data and a 4 mm diameter sphere was used to quantify average retention in the muscle. Initial uptake rates were calculated by curve fit of time-activity plots. Tumor-to-muscle ratios (T/M) were calculated by dividing the activity present in the tumor by the activity present in the muscle. The log of the ratio of T/M between Day 1 to Day 0 was also calculated as $\log_{10}\frac{\frac{T}{M}Day1}{\frac{T}{M}Day0}$. Blockade of retention would be indicated by a decrease in this value.

### 2.5. 2,4-Dinitrophenol and Pyruvate to Increase Oxygen Consumption Rate and Retention of [^18^F]FAZA

SK-MEL-5 tumors were grown to >100 mm^3^. 2,4-Dinitrophenol was freshly prepared and sterile filtered prior to experiments. After a baseline scan on Day 0, tumors were directly injected (i.t.) with 2,4-dinitrophenol or saline vehicle just prior to administration of [^18^F]FAZA on Day 1. Similarly, 200 μL of 100 mM sodium pyruvate (pH = 6.8; Sigma Aldrich) or saline was administered i.v. 30 min prior to [^18^F]FAZA injection on Day 1.

### 2.6. Pimonidazole Immunohistochemistry

Subsequent to [^18^F]FAZA injection and 3 h prior to euthanasia, mice were injected with 60 mg/kg pimonidazole HCl. Tumors were fixed for 24 h in neutral buffered formalin and then exchanged for 70% ethanol and processed for immunohistochemistry. Slices were scored by an independent histologist that did not have access to the PET data.

### 2.7. Human PET/CT Scanning

Prior to imaging patients, IND approval for clinical [^18^F]FAZA PET/CT (MDACC; IND#135054) was completed and a University of Texas MD Anderson Cancer Center Institutional Review Board-approved protocol activated (MDACC#2016-0847) for baseline [^18^F]FAZA PET/CT analysis. The IHC analysis was conducted under a University of Texas MD Anderson Cancer Center Institutional Review Board-approved and activated protocol (MDACC# 2012-0441). Patients provided informed consent and studies were conducted according to relevant guidelines and regulations. Patients were injected intravenously with 5.2 MBq/kg ±10% (10 mCi/70 kg) of [^18^F]FAZA and imaged at 60 min post injection using a GE 690 PET/CT scanner. The patients were first positioned supine in the scanner and a CT scout was acquired to determine the location of the area of interest in the patient. A low dose CT scan was then acquired for attenuation correction and anatomical registration over this area of interest. The CT scan parameters were 120 kV with tube current modulation, slice thickness/spacing = 3.75/3.27 mm, rotation speed = 0.5 s, pitch of 0.984 (resulting in 39.37 mm/rotation) and a detector configuration of 64 × 0.625. The CT scan was then followed by a single FOV PET scan over the same area of interest acquired in LIST mode for a duration of 180 min. At the end of the PET scan, a whole body (apex to toes) PET/CT scan was then performed. The PET LIST mode data were then rebinned into multiple 30 min scans (60, 90, 120, 150, 180, 210 min post injection). In the event that a patient required a break during the 3 h PET scan, the acquisition was halted and restarted again after reacquiring the necessary CT scan. All PET data, rebinned LIST mode and whole body scans were then reconstructed with data corrections for attenuation, scatter, randoms, and deadtime using standard manufacturer-supplied software. Image reconstruction was performed using ordered subsets expectation maximization (OSEM) techniques with 2 iterations, 18 subsets, 5 mm post filtering, using Time of Flight and resolution recovery into a matrix size of 192 × 192 and a field of view of 70 cm. The same process was also repeated the next day for the retest study. VOIs were then drawn on the resultant images using PMOD software. Control regions were defined as the contralateral side of the brain with the same size VOI except mirrored.

### 2.8. Statistics

When comparing two groups *t*-tests were utilized (GraphPad Prizm 6.01) [36]. When multiple comparisons were made post ANOVA, Sidak adjustments of *p*-value for multiple comparisons were utilized. Cohen’s *d* was calculated as the ratio of difference in the mean and the pooled standard deviation. Data are presented as mean ± SEM as default or ± SD as indicated in the text. IC_50_ values for OCR data were fitted using a four compartment model after normalizing to DMSO controls, *n* = 5 per dose. Dose-response curves in vivo were calculated utilizing a 4-compartment model, *n* = 3 per dose; for pimonidazole, fitting the baseline was constrained to 0 to yield convergence. In total, 95%CI were calculated based in log space and transformed into a linear %baseline.

## 3. Results

### 3.1. Inhibition of Oxygen Consumption in Cellulo

**Figure 2:**
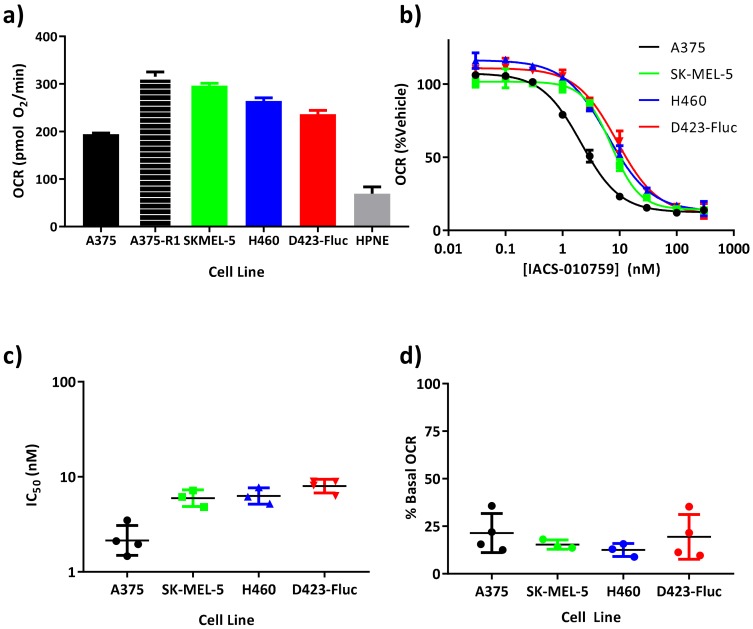
IACS-010759 is a potent nanomolar inhibitor of oxygen consumption rate in vitro. (**a**) Various tumor cells or HPNE cells were seeded 24 h prior to analysis of oxygen consumption rate (OCR; mean ± SEM; *n* > 136 wells; >3 biological replicates). Oxygen consumption rate of A375-R1 cells is included from the literature for completeness [29]. (**b**) After an initial oxygen consumption rate measurement, IACS-010759 was added and concentration-response curves were fitted to the data (mean ± SEM; *n* = 4 wells per concentration). (**c**) Independent curves were fit to each biological replicate yielding low nanomolar IC_50_ values for each cell line (mean ± SEM; *n* ≥ 3). (**d**) Residual oxygen consumption rate at 300 nM IACS-010759 was calculated from the curve fits and displayed for each replicate.

A panel of five tumor cell lines with various genetic, epigenetic, and biochemical mechanisms of metabolic dependencies on OxPhos were chosen for analysis of OCR. OCR was measured through extracellular flux analysis in a microwell plate (Seahorse, Agilent). D423-Fluc glioblastoma tumor cells are deficient in glycolysis through deletion of enolase-1, which is on the 1p36 tumor suppressor locus, sensitizing them to mitochondrial metabolic blockers [30,37,38]. A375 cells are mutant BRAF^V600E^ melanoma tumor cells that can be partially killed by BAY-87-2243, another complex-I inhibitor that is not clinically advancing [39]. After escape from MEK inhibitor treatment, the clonally-derived A375-R1 cells demonstrate elevated OxPhos and drug resistance to both MEK inhibitors and BRAF inhibitors in an OxPhos-dependent manner [28,29]. SK-MEL-5 melanoma cells are OxPhos dependent [29,39], mechanistically linked to biochemical alterations in iron metabolism and up-regulation of iron efflux that disrupts mitochondrial heme complexes and energetics, resulting in sensitivity to mitochondrial inhibitors [40,41]. H460 NSCLC cells are glutamine oxidation-dependent in monolayer culture, but switch to reductive glutamine metabolism via cytosolic isocitrate dehydrogenase 1 (IDH1) and oxidative metabolism under anchorage-independent conditions [42], consistent with human NSCLC in vivo [19]. While the tested tumor cells were all OxPhos-dependent through different mechanisms, under baseline conditions, A375, SK-MEL-5, H460 and D423-Fluc cells all consumed oxygen with similar basal rates (tumor OCR range; pmol O_2_/min: 194.6 ± 27 to 315 ± 20; *n* ≥ 30 wells per plate; *n* ≥ 3 biological replicates) as determined by a microplate oxygen consumption analysis (SeaHorse) (Figure 2a). The oxygen consumption rate of A375-R1 cells is included from the literature for comparison [29]. HPNE are h-TERT immortalized human pancreatic normal epithelial cells and by comparison yielded both the lowest OCR and the lowest OCR per seeded cell.

IACS-010759 exhibited nanomolar inhibition of oxygen consumption rates in all tumor cells (Figure 2b) and in immortalized normal cells (NIH3T3) [30]. IC_50_ values ranged between 2.1 nM and 7.9 nM (LogIC_50_ values between 0.33 ± 0.6 and 0.9±0.071; mean ± SD; *n* ≥ 3 titration curves). The IC_50_ of A375 tumor cells was lower than the other three tumor cell lines (*p* < 0.01, ANOVA post hoc multiple comparisons Sidak adjusted). Residual post-drug OCR was low, ranging between 12.5% ± 3.4% of control for H460 cells (Figure 2c) and 21% ± 10% of control for A375 cells (*p* ≤ 0.0006, for each cell line tested as difference from 100%) (Figure 2d). An additional experiment validated that the oxygen consumption rate of A375-R1 cells was inhibited by IACS-010759 with an IC_50_ of 4 nM and residual OCR of 24%, similar to the other tumor cell lines (Figure S1).

### 3.2. [^18^F]FAZA Retention in Tumor-Bearing Mouse Models: Reproducibility and Optimizing Analysis

**Figure 3:**
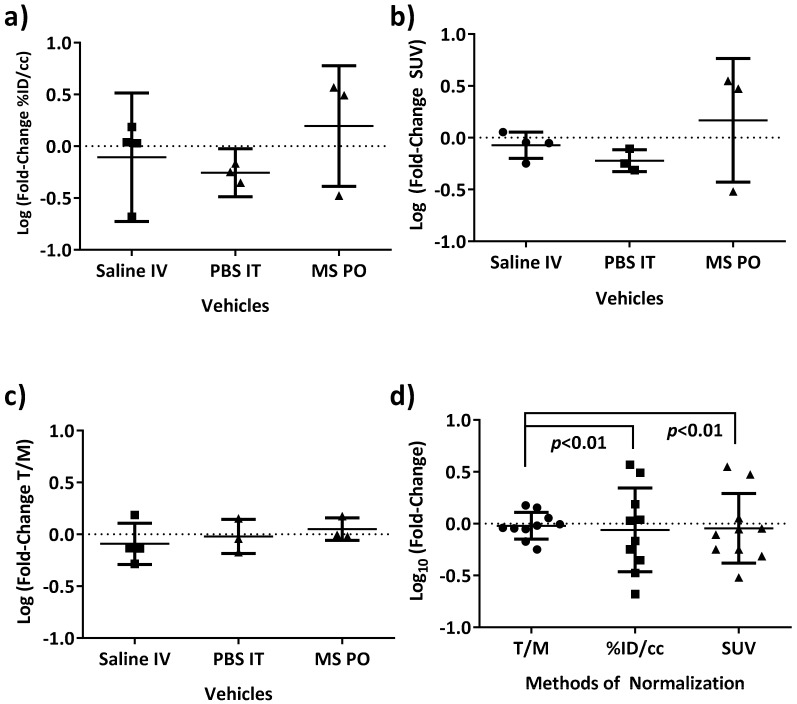
The log fold-change of [^18^F]FAZA PET T/M ratios yield well-centered and precise measurements for vehicle treatments of SK-MEL-5 tumor bearing mice. To establish precision for baseline analysis of data, vehicle treatments by various routes (i.v., i.t., p.o.) in SK-MEL-5 tumor-bearing mice across the study were examined in detail. A metric that was well centered at 0 value (null hypothesis) with precision was desired. After calculating the log fold-change of (**a**) %ID/cc, (**b**) SUV_mean_, (**c**) T/M, and (**d**) pooling all the vehicles, the log fold-change in tumor to muscle ratio (T/M) was selected as the highest precision metric for the remainder of the analysis.

Next, the hypoxia PET imaging agent, [^18^F]FAZA, was prepared by standard methods [43]. Using various routes of vehicle administration (i.v., i.t., p.o.) in mice with SK-MEL-5 flank tumors, PET images at 3 h post injection of [^18^F]FAZA were analyzed to determine which of several common image metrics yielded the highest precision (Figure 3a–c). Using a two-day test/retest methodology, a value well-centered at 0 was the expected null hypothesis in the log fold-change of standard calculated imaging metrics (e.g., %ID/cc, standardized uptake value (SUV_mean_), and tumor to muscle ratio (T/M)). T/M normalization yielded the smallest variance across vehicle-treated mice and was well-centered at 0 for day to day change, even accounting for the challenges of log-transformed data, which may be insensitive to small linear changes due to compression of data points. When all vehicle-treated data were pooled, log fold-change T/M ratios were still both well centered around 0 and yielded the smallest variances (*F*-test, *p* < 0.01) (Figure 3d). Therefore, the log fold-change in T/M ratio was utilized for the analysis of all experimental PET data sets.

### 3.3. Reversal of [^18^F]FAZA Retention In Vivo Across Multiple and Diverse Tumor Models Treated with IACS-010759

**Figure 4:**
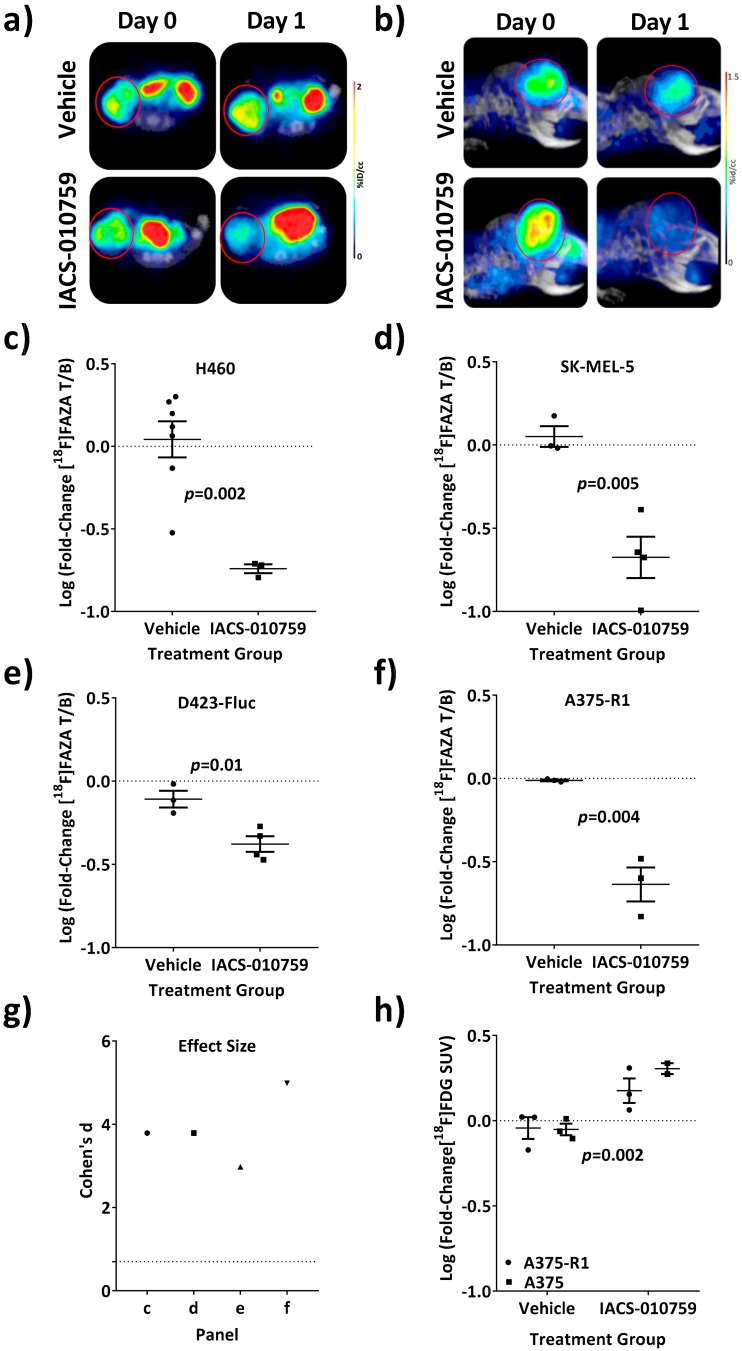
Animals treated at the MTD of IACS-010759 demonstrated a significant decrease in [^18^F]FAZA retention vs. vehicle-treated animals. Prior to treatment, mice bearing (**a**) H460 NSCLC subcutaneous tumors, or (**b**) orthotopic D423-Fluc glioblastoma tumors were injected i.v. with ~11 MBq of [^18^F]FAZA and imaged by PET/CT 3 h post injection in a small animal PET/CT scanner (Day 0). They were then treated p.o. with either vehicle or IACS-010759 (10 mg/kg) and re-imaged the next day (Day 1). Representative PET/CT images (transaxial slices and sagittal MIPs, respectively) are shown as %ID/cc and tumors circled in red for clarity. (**c**–**f**) NSCLC H460, melanoma SK-MEL-5, orthotopic glioblastoma D423-Fluc, and BRAF inhibitor-resistant A375-R1 tumors were imaged; differences between Day 0 and Day 1 were analyzed. VOIs were drawn using CT data and PET T/M ratios quantified. Each animal served as its own baseline control and the log fold-change in T/M was calculated. Easily detectable differences in [^18^F]FAZA retention after treatment with 10 mg/kg IACS-010795 (p.o.) were observed when compared with vehicle-treated animals in all cases (*p* < 0.01). While the magnitude of change was different for each tumor type, the direction of change was concurrent for all tumors. (**g**) Effect size describes the ability of a test to discriminate different populations; effect size for each tumor type was plotted and the calculated threshold for large effect size is included for reference (dotted line). (**h**) [^18^F]FDG PET/CT was performed before and after treatment with IACS-010759 (10 mg/kg) on A375 and A375-R1 tumor-bearing mice and the log fold-change in SUV_mean_ was quantified. As analyzed by two-way ANOVA, while highly precise and reproducible (vehicles were well centered around 0), there was indeed a detectable increase in [^18^F]FDG retention upon treatment with IACS-010759 that was smaller than the detectable decrease in [^18^F]FAZA (**f**).

A useful PET imaging pharmacodynamic marker should be robust and yield similar results across diverse models. We tested our methodology across both orthotopic and subcutaneous models as well as across diverse tumor types in both nude and NSG mouse strains. To quantify reversal of consumptive hypoxia, orthotopic glioblastoma (D423-Fluc), subcutaneous non-small cell lung cancer (H460), and orthotopic melanoma (SK-MEL-5 and A375-R1) models were imaged before and 24 h after treatment with a previously-determined maximal tolerated dose (MTD) of IACS-010759 in mice (Figure 4a,b). [^18^F]FAZA retention, as measured by log fold-change in T/M ratio, was significantly reduced at 24 h post-treatment. In *all* tumor models, retention of [^18^F]FAZA was robustly reversed at the MTD of 10 mg/kg (Figure 4c–f). The precision of the test-retest methodology in mice yielded large effect sizes for these tumors (Figure 4g), indicating that these would be suitable as an in vivo imaging assay. By comparison, changes in [^18^F]FDG SUV_mean_, a standard measure of glucose uptake and trapping, were modestly increased (~1.5-fold) in both A375 and A375-R1 tumors post-treatment with IACS-010759 (Figure 4h). In terms of relative magnitude of change, the modest [^18^F]FDG increase was far less than the typical five-fold decrease observed in [^18^F]FAZA T/M ratios.

### 3.4. Loss of [^18^F]FAZA Retention Post-Treatment with IACS-010759 Is Not Related to Changes in the Initial Uptake of [^18^F]FAZA

Two alternative explanations for the loss of [^18^F]FAZA retention upon treatment with IACS-010759 required exclusion. First, complete respiratory blockade and cell death by 24 h from a single dose of drug would abrogate production of NAD(P)H and thus obviate cellular trapping of [^18^F]FAZA. If this alternative mechanism explained the loss of [^18^F]FAZA retention, rapid and complete eradication of the tumor would also yield a complete loss in [^18^F]FDG uptake at 24 h; this was not observed (Figure 4h). The second alternative would be vascular collapse and a loss of delivery of small molecules such as [^18^F]FAZA to the tumor cell compartment. Again the increase in [^18^F]FDG retention does not support vascular failure. Vascular collapse would also predict that IACS-010759 treatment would result in both a loss of retention at 24 h and a similar loss of initial tracer uptake. A potential reduction of initial uptake was tested by treatment of a cohort of mice bearing SK-MEL-5 tumors, followed by dynamic PET imaging during initial in-flow, early retention (10 min), and final steady-state retention (3 h) of [^18^F]FAZA, both before and after treatment with IACS-010759. While data readily confirmed the reversal of [^18^F]FAZA retention at 3 h in response to IACS-010759, there were no detectable changes in either the initial uptake rate or the early retention of [^18^F]FAZA (Figure S2). Thus, the change in overall [^18^F]FAZA retention could not be explained by a simple reduction in vascular delivery impacting the input function.

### 3.5. Increasing [^18^F]FAZA Retention with Biochemical Drivers of Oxygen Consumption Rate

**Figure 5:**
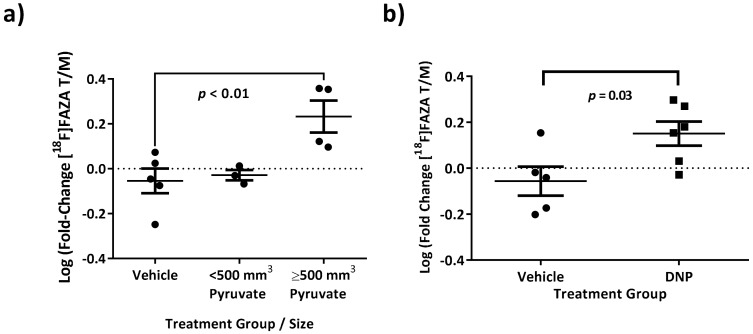
[^18^F]FAZA retention could be enhanced by small molecule interventions that increase oxygen consumption rate. SK-MEL-5 melanoma tumors were grown to a variety of sizes, and the log fold-change in [^18^F]FAZA retention was calculated for mice treated with (**a**) pyruvate, 200 µL of 100 mM solution i.v. or (**b**) 2,4-dinitrophenol (DNP, intra-tumor). Both were predicted to increase [^18^F]FAZA retention by accelerating oxygen consumption rate in the tumor. ANOVA analysis indicated that, in larger tumors, [^18^F]FAZA retention was increased with pyruvate when compared with vehicle control, but not in smaller tumors. Furthermore, there was a detectable increase in [^18^F]FAZA retention when tumors were treated with DNP (*t*-test).

As further validation of the mechanism by which IACS-010759 reversed [^18^F]FAZA retention, one would hypothesize that the converse experiment, *enhancing* oxygen consumption rate, should concomitantly increase [^18^F]FAZA retention through increased hypoxia/reduction potential. This was tested with small molecule inducers of oxygen consumption rate, 2,4-dinitrophenol (DNP), a known protonophoric mitochondrial uncoupler, and pyruvate, an intermediate metabolite that feeds OxPhos via the TCA cycle. DNP was injected intra-tumorally at doses known to be tolerated by animals [44], yielding a hypothetical intratumor concentration of ~500 μM based on CT volumes of tumor masses calculated from pre-injection images immediately prior to treatment (Day 0). Pyruvate was injected i.v. following a literature protocol [45]. Both DNP (*p* = 0.03, *t*-test, two-tailed) and pyruvate in larger (substrate-limited) tumors (*p* = 0.01, ANOVA multiple comparison to vehicle, Sidak correction) yielded statistically detectable increases in [^18^F]FAZA retention in vivo (Figure 5). Therefore, overall, both inhibitors (IACS-010759) and enhancers (DNP and pyruvate) of oxygen consumption rates, respectively, yielded the predicted quantitative changes in [^18^F]FAZA retention in vivo.

### 3.6. Dose-Response of IACS-010759-Mediated Reversal of [^18^F]FAZA Retention in a BRAF Inhibitor-Resistant Melanoma Tumor Model In Vivo

**Figure 6:**
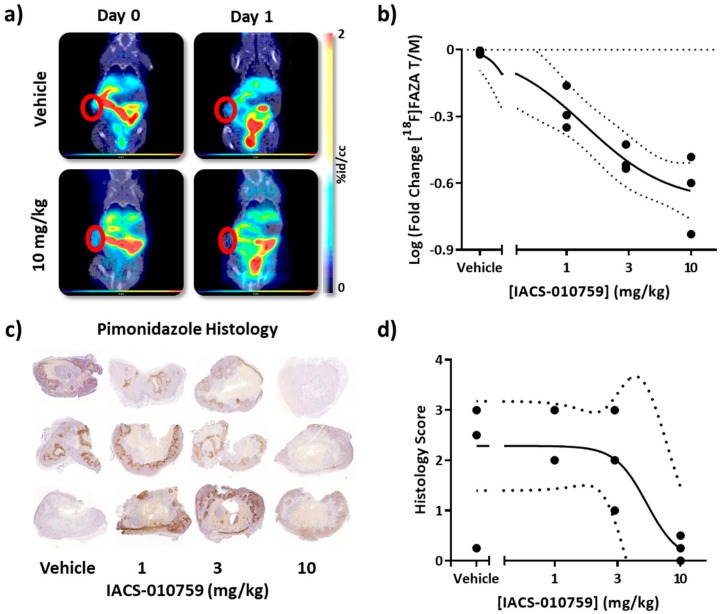
[^18^F]FAZA retention by PET/CT yielded a quantitative IC_50_ value similar to histological markers of hypoxia but with enhanced precision in the A375-R1 melanoma model. BRAF inhibitor-resistant A375-R1 tumors were grown subcutaneously and imaged with [^18^F]FAZA PET/CT. After baseline imaging (Day 0), mice were treated with vehicle or 1, 3, or 10 mg/kg IACS-010759 p.o. and 24 hrs later re-imaged (Day 1). Moreover, 3 h prior to euthanasia, all mice were injected with pimonidazole HCl (60 mg/kg) i.p. and tumors processed for IHC. (**a**) Example coronal images of mice treated with vehicle or IACS-010759 (10 mg/kg) from Day 0 and Day 1. (**b**) PET/CT data were quantified, log fold-change in T/M plotted vs. delivered dose, and fitted to a four parameter dose-response curve. (**c**) Pimonidazole IHC slices from all twelve mice were scored by an independent pathologist. (**d**) IHC scores were also fitted to a four parameter dose-response curve yielding similar IC_50_ values, but less precision (dotted lines indicate 95% CI of the fit).

To establish [^18^F]FAZA as a molecular-specific pharmacodynamic marker of target engagement in vivo, A375-R1 tumor-bearing mice were baseline pre-imaged with [^18^F]FAZA, treated with IACS-010759 at four different doses on Day 0 (0, 1, 3 and 10 mg/kg PO) and imaged again 24 h later (Day 1), followed by injection with pimonidazole (Hypoxyprobe) as a conventional ex vivo gold standard of hypoxia and the validated PD biomarker for IACS-0101759 (Figure 6). [^18^F]FAZA retention was readily observed and for vehicle-treated mice, the % change from Day 0 to Day 1 was 2.7% ± 1.9% (mean ± stdev).

A clear dose-response of IACS-010759-induced reversal of [^18^F]FAZA retention could be observed non-invasively by PET imaging in vivo. Semi-quantitatively, a similar dose-response was recorded with pimonidazole IHC in the same cohort ex vivo. Both methods yielded comparable IC_50_ values ([^18^F]FAZA PET, 1.4 mg/kg vs. pimonidazole, 5 mg/kg). However, in this model, the repetitive test-retest [^18^F]FAZA PET imaging yielded a more precise measurement of the IC_50_ compared to pimonidazole: 95% CI: 0.5–3.5 mg/kg, *R*^2^ = 0.88; versus 95% CI: 1.5–19 mg/kg, *R*^2^ = 0.5, respectively (4 compartment model with Hill slope). This could be due to not only the sampling limitation, but also the significant difference in dynamic range and linearity of response that would be expected for scored IHC when compared with immunofluorescence (IF) [46] or quantitative PET.

### 3.7. Precision of [^18^F]FAZA PET/CT Test-Retest Method in a Patient with Glioblastoma

**Figure 7:**
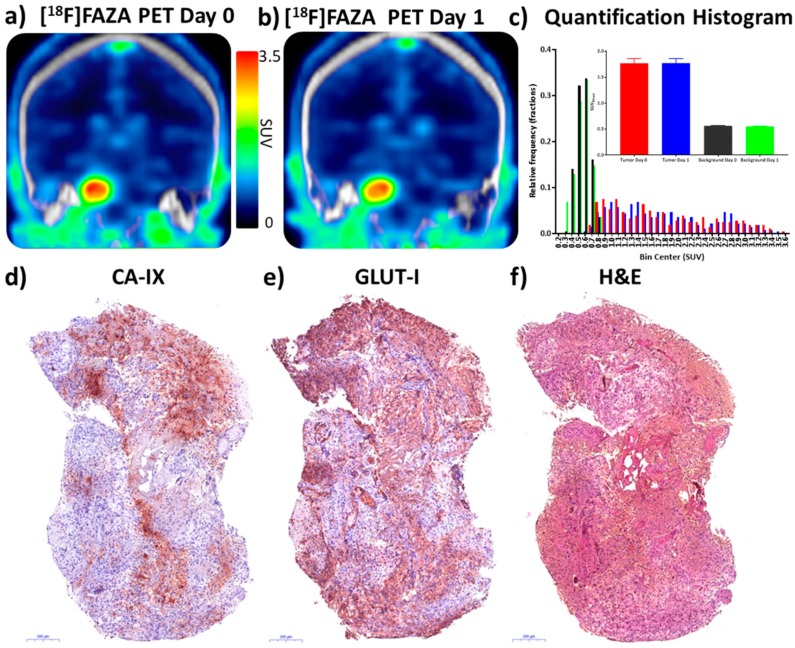
[^18^F]FAZA retention in a patient with glioblastoma yields high contrast, reproducible PET/CT images of hypoxia confirmed by immunohistochemical analysis. A patient with a grade IV right pontine glioblastoma was enrolled in an ongoing test-retest clinical trial and injected with [^18^F]FAZA (5.2 MBq/kg) on Day 0 and Day 1. PET/CT scans were acquired in LIST mode for analysis. (**a**, **b**) PET/CT images 2 h post injection were reconstructed and converted into SUV units (g/cc); representative coronal images through the peak of tumor retention are illustrated. (**c**) VOIs were drawn from PET images and background VOIs were created by mirroring tumor VOIs to contralateral brain. Individual pixel values were extracted from VOIs and histograms plotted of Day 0 and Day 1 pixel values for both tumor and background; (inset) SUV_mean_ ± 95% CI. Key for c: Tumor Day 0 = red, tumor Day 1 = blue, background Day 0 = black, background Day 1 = green. (**d**, **e**) Post-op tumor specimens were stained for two independent markers of cellular hypoxia, CA-IX and GLUT-1. (**f**) H&E specimen.

All institutional IND and IRB approvals were obtained for a proof-of-principle pilot human study aimed to consolidate the precision of the test-restest method in two consecutive days and provide the baseline for a future clinical trial where [^18^F]FAZA PET/CT was used as PD marker for IACS-01079 in patients. A patient with a biopsy-proven, untreated 2.5 cm right pontine grade IV glioblastoma tumor (IHC: IDH1 WT; p53 scattered positive; ATRX WT) was imaged by [^18^F]FAZA PET/CT in two consecutive days, as proof of precision of the test-retest methodology (Figure 7a,b).

A patient with glioblastoma was selected for imaging and analysis because glioblastoma tumors tend to be hypoxic, radio-resistant, and are reported to retain another hypoxia PET tracer, [^18^F]FMISO. They are therefore likely to be [^18^F]FAZA positive [47,48,49,50]. Images were converted to SUV and tumor and background (contralateral volume) retention values were measured on Day 0 and Day 1 at 1 and 2 h post injection of [^18^F]FAZA. SUV_mean_ and log fold-change values were calculated. Day 0 SUV_mean_ values were 1.766 and 0.561 for tumor and background (2 h). Day 1 SUV_mean_ values were 1.772 and 0.544, respectively. The % change values from Day 0 to 1 were 0.3% and –3.0% and log fold-change values were 0.0015 and –0.013. All remaining data values are presented in Figure S3. Radiomic histograms of pixel-by-pixel tumor and background intensities were highly reproducible as was the SUV_mean_ in both tumor and background regions (Figure 7c). Thus, in a test-retest analysis of [^18^F]FAZA PET/CT as a potential pharmacodynamic marker, baseline analysis of the null hypothesis showed changes in human tumor retention of the tracer of <1%, exceeding the precision of pre-clinical baseline animal studies. Direct IHC analysis of the post-surgical specimens showed robust staining of CA-IX and GLUT-1 (Figure 7d–f), both downstream targets of HIF-1α, independently confirming the presence of tumor hypoxia.

## 4. Discussion

Normal cells readily switch between OxPhos and glycolysis [51,52,53]. Either genetic or pharmacological inhibition of OxPhos results in compensatory up-regulation of glycolysis to maintain ATP levels, resulting in only modest inhibition of proliferation [54]. In effect, normal glycolysis is under negative control by OxPhos, via tricarboxylic acid cycle (TCA)-mediated allosteric inhibition of glycolysis enzymes, i.e., the “Pasteur effect” [55]. Mechanistically, inhibition of OxPhos forces dependence on glycolysis, which is tolerated in normal cells because they can up-regulate glycolytic enzymes, but is not sustainable in subclasses of tumors that have insufficient capacity to up-regulate glycolysis and/or have limited production of mitochondrial-dependent essential metabolites, such as aspartate, to meet their high metabolic and growth demands [30]. Other tumors may have genetic defects, such as enolase-1 (ENO1) deletion, which prevent ENO1-deleted tumors from upregulating glycolysis in response to OxPhos inhibition [30,37,38]. Thus, the OxPhos dependency in these tumor subsets versus the metabolic plasticity of most normal cells provides a potential vulnerability for molecularly targeted therapy and an actionable therapeutic window for candidate mitochondrial complex-I inhibitors, such as IACS-010759.

Hypoxic tumor cells utilize HIF-1, a transcription factor, to regulate cellular metabolism, anabolism, and proliferation in response to hypoxic conditions [14]. HIF-1 is well known to induce enzymes responsible for enhanced glycolysis under conditions of low oxygen and has also been reported in RCC cells to actively repress mitochondrial oxygen consumption rates by between two- and three-fold, resulting in a relative increase in intracellular oxygen tension by upregulating pyruvate dehydrogenase kinase 1 (PDK1) [1]. Pyruvate dehydrogenase (PDH), which irreversibly converts pyruvate to acetyl-CoA [56], is inhibited when phosphorylated by PDK1, leading to *inhibition* of pyruvate flux into the TCA cycle within mitochondria [1], and thereby favoring pyruvate conversion to lactate by LDH, regeneration of NAD^+^, and secretion of lactate. Thus, the NAD^+^ cycle and redox state of the cell are proposed to be maintained through this coupled system coordinated by HIF-1 [1,57], and the shift in glucose [9] and intermediate metabolism away from OxPhox thus provides a resourceful means for the cell to maintain both energy flux and redox state [58]. However, somewhat paradoxically, measured mitochondrial O_2_ consumption rates in hypoxia remain quite significant even under conditions of severe hypoxia (pO_2_ levels of ~0.5 mm Hg) [10].

Our goal was to test the hypothesis that PET/CT imaging with [^18^F]FAZA, a second-generation 2-nitroimidazole radiopharmaceutical, could quantitatively monitor the inhibition of OxPhos in vivo by imaging the reversal of consumptive (demand) hypoxia as a repetitive, non-invasive PD marker of IACS-019759 in living subjects. [^18^F]FAZA has been robustly validated as a hypoxia imaging agent through both oxygen sensor experiments and MRI correlation studies in preclinical tumor models [31,59] and shows superior blood clearance profiles in mice when compared to other PET agents, such as [^18^F]F-MISO, resulting in greater tumor to background ratios [31,60]. Complex-I, which is upstream of oxygen consumption (complex IV), contributes a majority of the electrons to the electron transport chain (ETC) per turn of the TCA cycle, and can be rate limiting for oxygen consumption [61]. While there have been attempts to measure OCR in deep tissue directly with ^17^O MRS/MRI [62], this technique is currently in preclinical development and the instrumentation and software are not readily available for clinical translation. Thus, [^18^F]FAZA and other nitroimidazole reporters, are candidate mechanism-based pharmacodynamic (PD) markers of complex-I inhibitors in living animals in vivo through the tight coupling of complex-I to complex IV and therefore oxygen consumption [4,63]. Furthermore, while [^18^F]FAZA PET imaging has been surveyed in patients, including in longitudinal studies [64,65,66], quantitative features such as test-retest precision and use as a quantifiable PD biomarker remain to be explored.

In tumor cells reliant on OxPhos, drugs that target complex-I would ultimately block electron transport and therefore decrease the consumption rate of oxygen, reverse hypoxia, yielding an oxygenated and less reductive subcellular micro-environment, consequently decreasing retention of [^18^F]FAZA. Herein, we provide quantitative analysis of changes in [^18^F]FAZA retention induced by the novel complex-I inhibitor IACS-010759 across a spectrum of metabolically reprogramed tumors. Importantly, these data lend support to a more general metabolic model of consumptive (demand) hypoxia driven by high OxPhos in many tumors [13].

First, as expected, IACS-010759 produced nanomolar inhibition of oxygen consumption rate *in cellulo* in a microwell plate assay (Seahorse) across the tumor cell lines tested, regardless of their mechanism of dependence on OxPhos. Furthermore, IACS-010759 treatment was able to abrogate between 85% and 90% of the initial oxygen consumption rate across the cell lines, regardless of the specific mechanism that enhanced OxPhos.

Using a rigorous test-retest protocol to determine the change in [^18^F]FAZA retention as measured by the log fold-change in the tumor to muscle ratio (T/M), there was sufficient precision to demonstrate full-target engagement in rodent tumor models near the expected MTD, as well as generate precise dose-response curves in vivo. The methodology accounted for tumor heterogeneity, variable vascular input functions, and systemic variations in reproducibly to yield data consistent across different tumor types, each with divergent underlying mechanisms of OxPhos dependence, from melanoma to lung cancer to glioblastoma. Such dose-response curves might be utilized to predict the effectiveness of IACS-010759 or other complex-I inhibitors against new tumor types once the maximum tolerated dose in humans is fully resolved.

Two trivial explanations for the drug-induced decrease in [^18^F]FAZA retention were excluded. First, single dose IACS-010759 did not immediately kill the entire tumor within the first 24 h. Herein, [^18^F]FDG retention not only served as a tumor viability marker, but interestingly, a statically significant IACS-010759-induced increase in [^18^F]FDG retention was observed in these particular tumors, consistent with the Pasteur effect. This was similar to the results from another OxPhos inhibitor study wherein [^18^F]FDG retention remained high and trended higher at 24 h post treatment, but did not yield statistically detectable differences [63]. Second, the possibility that the IACS-010759-induced decrease of [^18^F]FAZA retention was due to compromised delivery of small molecules (such as [^18^F]FAZA *per se*) following treatment was ruled out by dynamically evaluating the initial delivery of [^18^F]FAZA into the tumor both before and after treatment. The [^18^F]FAZA retention at 10 min p.i. before or after treatment was not decreased, confirming that delivery of small molecules was uncompromised, whereas the anticipated IACS-010759-induced decrease in [^18^F]FAZA retention was observed at 3 h p.i. By extension, it is likely that overall perfusion of the tumor was also not compromised at 24 h. Indeed, in a previously reported kinetic analysis of [^18^F]F-MISO trapping, heterogeneous regions of tumor were observed with both high and low perfusion (as measured by K1), but *both regions* had low oxygen content and rapid [^18^F]F-MISO trapping [67]. In those regions of high delivery but low oxygen, one might speculate that a high oxygen consumption rate caused the low net oxygen content and rapid trapping of [^18^F]F-MISO. However, even this explanation may yet be too simplistic. While flow rates are captured, microscopic velocities, particularly directionality, are not captured. For example, in 4T1 breast tumors, areas of high flow velocity leaving a hypoxic area can lead to further loss of oxygen and exacerbate local hypoxia [68]. Furthermore, it was recently reported that in GBM patients, high nitroimidazole retention and CA-IX staining co-correlated with contrast enhancement, and thus, simply invoking lack of delivery is insufficient to explain local hypoxia [48], further re-enforcing the model that both the delivery and consumption rate of oxygen contribute to the net hypoxic/redox state of tumors [13]. Thus, *in toto,* it is unlikely that IACS-010759 impacted [^18^F]FAZA trapping by changing the delivery of oxygen to the tumor, but rather by changing oxygen consumption rates.

We further documented the mechanisms of [^18^F]FAZA retention by predictably *enhancing* hypoxia though small molecule interventions that increase oxygen consumption rate. Two well-studied small molecule modulators of oxygen consumption rate, 2,4-dinitrophenol (DNP) and pyruvate, were employed. They should act by creating a more hypoxic microenvironment through different, but related, biochemical mechanisms. First, complex-IV consumes oxygen and pumps additional protons against the electrochemical gradient between the inner mitochondrial matrix and the inter-mitochondrial membrane space. DNP acts as an uncoupler, shuttling protons across the inner mitochondrial membrane to dissipate this gradient, thus maximizing oxygen consumption rates in the cell for a given flux of electrons down the ETC. By comparison, pyruvate drives the TCA cycle through mass action, increasing electron flux through the ETC, and increasing oxygen consumption rates. Both 2,4-dinitrophenol and pyruvate yielded the predicted increases in [^18^F]FAZA retention compared to vehicle-treated mice, confirming that interventions within mitochondria could modulate intracellular oxygen availability in directions opposite to inhibition of complex-I (Figure 5). In SK-MEL-5 tumors greater than 500 mm^3^, there was an increase in [^18^F]FAZA retention after i.v. injection of pyruvate, similar to data reported in models of colorectal cancer using pimonidazole [45]. As expected, SK-MEL-5 tumors also responded to IACS-010759 inhibition of OxPhos (Figure 4d). These data corresponded with literature data wherein increases in retention of [^18^F]FAZA have been reported upon treatment with a non-specific inhibitor of glycolysis, dichloroacetic acid (DCA), and indirect inhibitors of glycolysis, such as EGFR inhibitors [69,70]. Based on our and prior data, one might speculate that other metabolic changes to the cell that shift the redox balance, such as fatty acid metabolism and oncogene expression, might well also impact [^18^F]FAZA retention.

Note that a necessary first step for assessing the final response of tumors to mechanism-based targeted therapy is documentation of target engagement, i.e., a PD marker. In particular, in the evaluation of new therapeutic agents, it is important to demonstrate that target engagement has been achieved at or below the maximum tolerated dose (MTD), and in the case of an inhibitor, that the target is blocked, thus providing the potential for efficacy [71]. PD markers are not prognostic or predictive biomarkers and correlation with long term patient survival or with drug-resistant versus drug-sensitive tumor states are outside of the scope of a PD study [72] (See Supplementary Materials). PD biomarkers ultimately can improve patient outcomes through their robust negative predictive power for linear mechanism-based targeted therapies. Drug developers can triage poor compounds early in clinical trials and individual patients can be switched off of a failing therapy early and onto more promising therapeutics or trials.

Critically, a dose escalation study was conducted to cross-validate the non-invasive [^18^F]FAZA PET/CT imaging with an already established invasive PD biomarker for IACS-010759, pimonidazole. Pharmacodynamic dose-response curves were generated in vivo with [^18^F]FAZA PET/CT imaging, yielding higher precision than immunohistochemistry with pimonidazole. While both datasets yielded a comparable IC_50_, 95% CI values as assessed by repetitive PET/CT were substantially smaller (0.5–3.5 mg/kg vs. 1.5–19 mg/kg). Pimonidazole IHC, while providing spatial resolution at the cellular level by visual analysis and agreeing broadly with the [^18^F]FAZA data in this model, nonetheless was not as precise as repetitive whole body PET/CT. [^18^F]FAZA analysis delivered additional precision and power likely derived from the ability to non-invasively resample the molecular physiology of the *identical* individual from day to day as well as the capacity to exam the entire tumor volume, thereby avoiding errors due to random sampling inherent to the biopsy techniques. Furthermore, IHC-based pimonidazole staining suffers from a compression of dynamic range and loss of linearity when compared with either immunofluorescence or PET. This arises from a product based stain for the trapped tracer rather than the one-to-one correspondence of the trapped radiotracer-nitroimidazole conjugate in PET or fluorophore conjugate in semi-quantitative immunofluorescence. Additionally, the more hydrophobic nature of pimonidazole and the weakly basic side chain decreases the dynamic range/pO_2_ responsiveness of the reporter [46].

Furthermore, these data enabled us to begin to parse the relative contributions of random resampling versus whole tumor sampling to improve precision. In a direct comparison with a cross sectional immunohistochemistry experiment, when just the second [^18^F]FAZA imaging session was utilized in the analysis, the 95% CI of the IC_50_ was 0.2 to 5.7 mg/kg, *R*^2^ = 0.6. Although the precision is further improved utilizing the test-retest methodology to normalize each animal to its own baseline, clearly sampling the whole tumor in vivo yielded the largest improvement in precision, when compared to immunohistochemistry. The final clinical value of non-invasive repetitive PET/CT imaging is likely underestimated, since herein inbred mice and a single class of tumor were utilized in this dose-response analysis. In the clinic, both the germline and somatic landscapes of patients and tumors will be heterogeneous and stochastic, and therefore, the variability in the baseline drug metabolism and excretion across individuals and tumor types would be expected to be greater [73]. Repetitive biopsies are challenging; the operator cannot truly resample the same location over time, and samples are limited to small sub-segments of the entire tumor, known to be heterogeneous for markers of hypoxia [74]. Furthermore, in a patient with multiple metastatic sites, PET/CT enables the sampling of all sites, while biopsy sampling is limited.

To inform the clinical potential of this PET PD biomarker, we undertook a pilot patient imaging study to assess whether the test-retest methodology would be sufficiently reproducible in humans to justify moving forward with a full baseline and PD analysis of IACS-010759. A female patient with a stage IV IDH-wild type glioblastoma was selected. The subject also underwent a right posterior fossa craniotomy and pontine biopsy, enabling the collection and staining of tumor tissue. The injected dose of tracer was adjusted to account for acceptable radiation doses to the patient, and both 1- and 2-h post injection PET scanning times were evaluated. No substantial changes in image content between 1- to 2-h images were measured other than continued clearance of tracer from the blood pool. Upon repeat injection and imaging 24 h later, patient data were highly reproducible, with < 1% change in tumor SUV_mean_ and < 1% change in tumor to background ratio (Figure S3). The >three-fold tumor to background ratio (T/B) in human was similar to the murine glioblastoma model, and both the day-to-day % change and log fold-change actually were less than the variance measured in murine models, which was encouraging for moving forward. Furthermore, IHC analysis of the tumor revealed robust staining of both CA-IX and GLUT-1, well established protein markers of hypoxia. While the spatial staining pattern of CA-IX and GLUT-1 were non-identical but overlapping, protein was clearly present and robust in the tumor specimen, consistent with [^18^F]FAZA retention. These results were similar to those observed in NSCLC [75] and differ from the lack of association of the biomarker in laryngeal squamous cell carcinomas [76]. Further study of these biomarkers in glioblastoma may be informative.

Nonetheless, there are limitations to our analysis. Because changes in [^18^F]FAZA retention require a decrease in signal, first and foremost a tumor must retain [^18^F]FAZA more effectively than the local background for this PET technique to be applied. This alone necessitates the test-retest methodology. The first test not only sets the individual baseline but confirms that the intervention experiment is possible. Because of the broad availability of PET/CT and PET/MRI, tumor volumes of interest can often be defined from the orthogonal anatomical information contained in the CT or MRI. Therefore, it is feasible that changes in tumor retention that are close to, and even below, the local background retention of [^18^F]FAZA can be measured, although with a limited dynamic range and sensitivity. Finally, and again inherent to the process, this technique requires two PET/CT imaging sessions with the associated dosimetry, which must be weighed against the risk/benefit of repetitive biopsies.

There are broader implications for the biochemical data contained herein. The change in intracellular oxygenation/reduction potential at 24 h from a single dose of IACS-010759 suggested that the drug might act as a radiosensitizer to enhance the effect of low linear energy transfer (LET) radiation, such as external beam therapy, proton therapy, and neutron therapy, as well as targeted radiotherapy, particularly with beta-emitters [77,78]. Furthermore, because it has been demonstrated that both activators and inhibitors of oxygen consumption rates can be measured by [^18^F]FAZA PET/CT, in addition to complex-I inhibitors, the PD profile of other drugs that might modify oxygen consumption rates might also be interrogated by [^18^F]FAZA. Indeed, a clinical trial was recently reported underway wherein [^18^F]FAZA PET/CT will be utilized as a biomarker for tumor re-oxygenation by metformin and compared with outcomes in radiation and chemotherapy (NCT02394652). Another clinical trial is reported to study the change in oxygen consumption rate in tumors via [^18^F]EF5 PET when treated with nelfinavir, an antiviral that also inhibits tumor oxygen consumption rate through an unknown mechanism [79]. Finally, there are emerging compounds that target complex-IV that might be interrogated via repetitive [^18^F]FAZA imaging [80,81].

The data herein indicated the potential for further study of [^18^F]FAZA PET, not only as a pre-clinical PD biomarker, but also as a clinical PD biomarker of complex-I inhibitors broadly. The broad range of tumors tested in both subcutaneous and orthotopic models, both herein and in prior work with other complex-I inhibitors, illustrate the robustness and potential for translation of this method to the clinic. [^18^F]FAZA PET has already been utilized in the clinic for qualitative observational analysis of several classes of tumors that are likely to be hypoxic, including lung, head and neck, and pancreatic tumors [82,83,84]. All components herein have active IND files such that quantitative test-retest analysis with [^18^F]FAZA PET/CT in a similarly designed clinical study will be attempted.

## Supplementary Materials

The following are available online at https://www.mdpi.com/2073-4409/8/12/1487/s1, Figure S1: Oxygen consumption in A375-R1 melanoma cells; Figure S2: IACS-010759-induced reduction in [^18^F]FAZA retention cannot be explained by a decrease in early delivery of [^18^F]FAZA; Figure S3: Pilot human glioblastoma study, T/B ratios and SUVmean; Supplemental Discussion.
