# Supplementary material for: Mechanism-Specific Pharmacodynamics of a Novel Complex-I Inhibitor Quantified by Imaging Reversal of Consumptive Hypoxia with [18F]FAZA PET In Vivo
Source: Cells. 2019 Nov 21;8(12):1487. doi: 10.3390/cells8121487 (PMC6952969; doi:10.3390/cells8121487)
Supplement: Supplementary file 1 [file cells-08-01487-s001.pdf]

Supplemental Figure S1.

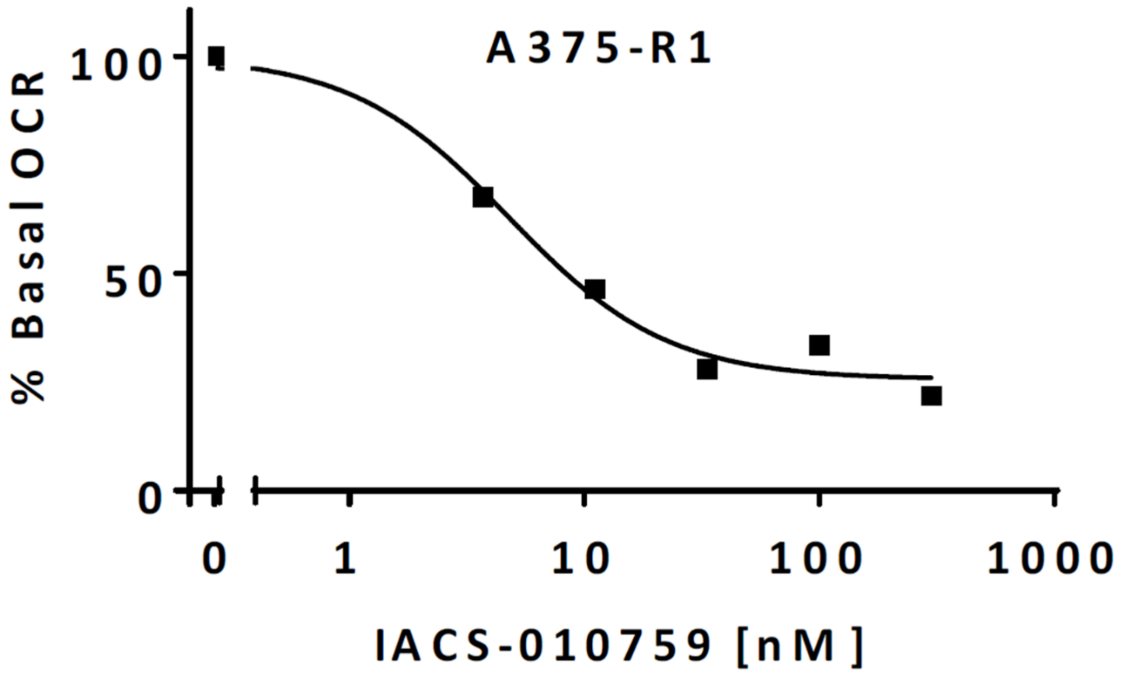

Figure S1. Oxygen consumption in A375-R1 melanoma cells can be inhibited by IACS-010795 with nanomolar IC_{50}.

A concentration-response curve for IACS-010759 was generated with A375-R1 cells tested in duplicate to confirm single nanomolar inhibition of oxygen consumption rate (OCR; Seahorse). Data were fit to a 4 compartment model yielding an IC_{50} of 4 nM and a residual oxygen consumption rate of 24% of baseline. This was consistent with the cell lines tested in Figure 2b and other human cell lines tested (Nat. Med. 24, 1036-1046, 2018).

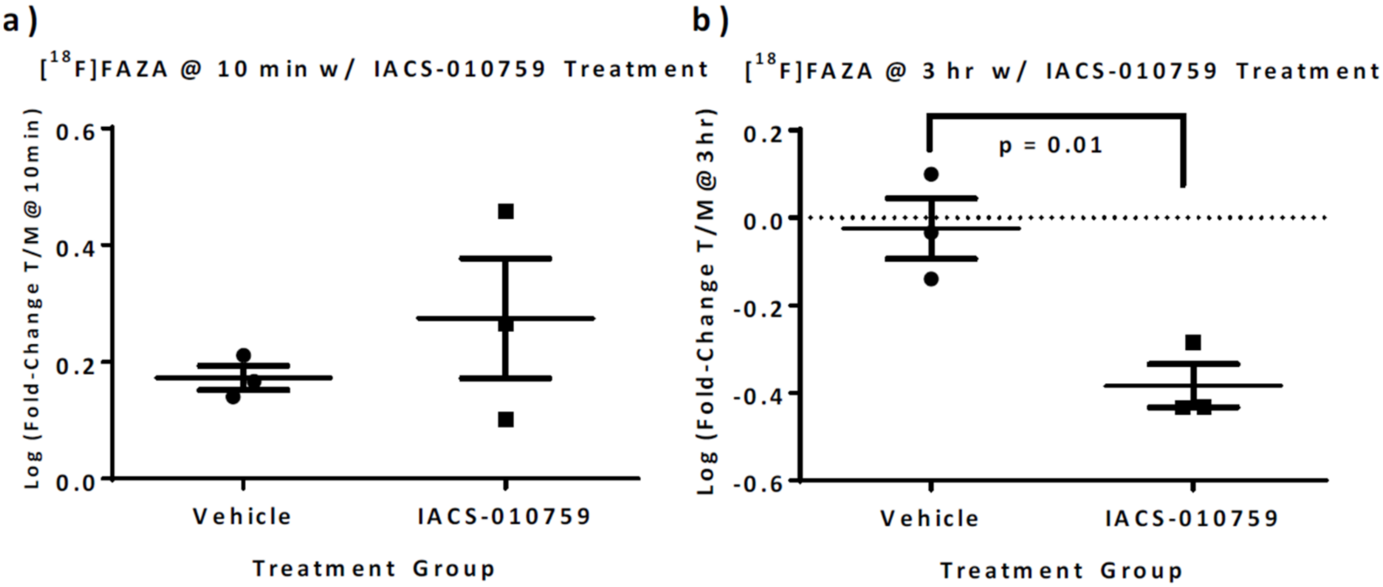

Figure S2. The significant IACS-010759-induced reduction in [^{18}F]FAZA retention at 3 hour post injection cannot be explained by a decrease in early delivery of [^{18}F]FAZA.

(a) Partial kinetic scans (10 min of dynamic scanning) immediately following bolus injection of [18F]FAZA, followed by 2 hour 50 minutes of clearance while animals were awake and ambulatory, and then a second 10 min PET/CT scan at 3 hours were acquired for A375-R1 tumor-bearing mice prior to treatment. Mice were treated with IACS-010759 (10 mg/kg) or vehicle and rescanned with the same protocol the next day. At 10 min p.i. (plateau of the initial uptake), there was no significant change in [^{18}F]FAZA uptake before and after treatment, and in fact, the trend was for a slight increase in the initial uptake for treated animals vs vehicle. However, the 3 hour p.i. time point from the same animals confirmed the expected IACS-010759-induced decrease in [^{18}F]FAZA retention ($p = 0.01$). These data, coupled with the [^{18}F]FDG data, indicated that loss of perfusion/delivery could not explain the decrease in [^{18}F]FAZA retention. p.i., post injection.

1 hour Relative Ratio

Metric	Day 0	Day 1	% Change	Lfc
T/B	3.38	3.37	-0.3	-0.0013

2 hour Relative Ratio

Metric	Day 0	Day 1	% Change	Lfc
T/B	3.15	3.25	3.2	0.013

1 hour SUV_{mean}

Metric	Day 0	Day 1	% Change	Lfc
Tumor	1.146	1.156	0.87	0.004
Background	0.339	0.343	1.2	0.005

2 hour SUV_{mean}

Metric	Day 0	Day 1	% Change	Lfc
Tumor	1.766	1.772	0.3	0.00147
Background	0.561	0.544	-3.0	-0.013

Figure S3. In a pilot human glioblastoma study, both T/B ratios and SUV_{mean} were highly reproducible at both 1 hour and 2 hour post injection of [^{18}F]FAZA.

Further quantification of the tumor in Figure 8 yielded both highly reproducible tumor to background ratios (T/B) and SUV_{mean}. In the brain, sufficient clearance of the tracer background may enable 1 hour imaging, but in the rest of the body, other optimal time points may be identified. Lfc, log fold-change.

Supplemental Discussion:

A first step in the preclinical/clinical development of a new compound is validation of a robust pharmacodynamic (PD) biomarker for use *in vivo* (*Biomark Med* **1**, 399-417, 2007). The primary goal of a PD biomarker is to demonstrate, ideally in a single dose, that the compound has reached and engaged the molecular target, a necessary (but insufficient) step toward proof-of-mechanism efficacy. Note that the goal of a PD biomarker is distinct from a predictive biomarker or a prognostic biomarker. Predictive biomarkers attempt to predict the final positive efficacy of a given compound, which can be complex. Efficacy can be complicated by signaling feedback loops, off-target drug effects and drug-resistant pathways. In the case of collateral lethality, for example, predictive biomarkers would likely exist downstream of target engagement or may require both target engagement and documentation of lack of relevant compensatory processes. By contrast, prognostic biomarkers are utilized to stratify patient survival and risks to patients prior to therapeutic interventions. As a special case, PD biomarkers are useful as negative predictors in preclinical studies once the human maximum tolerated dose (MTD) is known and may be useful for personalized dosing in patients. For example, for molecular-targeted therapy in patients, if a threshold for target engagement is not achieved as assessed by the PD biomarker prior to the individual patient exhibiting dose limiting toxicity, then the patient might be switched to an alternative therapy.
